# Melatonin Enhances Growth and Glucosinolate-Associated Nutritional Quality of Mustard Sprouts Under Moderate Salinity Stress

**DOI:** 10.3390/plants14233553

**Published:** 2025-11-21

**Authors:** Xiaoling Zhao, Xuena Yu, Hongmei Di, Aolian Zhou, Zhongrong Guan, Pingping Shi, Sen Wang, Bo Sun

**Affiliations:** 1 College of Horticulture, Sichuan Agricultural University, Chengdu 611130, China; zhaoxiaoling@caas.cn (X.Z.); 80251@sicau.edu.cn (X.Y.); 2023105007@stu.sicau.edu.cn (H.D.); 2022305050@stu.sicau.edu.cn (A.Z.); 2 Institute of Urban Agriculture, Chinese Academy of Agricultural Sciences, Chengdu 610213, China; 3 Southeast Chongqing Academy of Agricultural Sciences, Chongqing 408000, China; cqflgzr@163.com (Z.G.); own2@163.com (P.S.)

**Keywords:** salt stress, melatonin, mustard sprouts, antioxidant capacity, glucosinolates

## Abstract

Salt stress profoundly affects plant growth and metabolism, whereas melatonin has emerged as an effective regulator that modulates plant responses to abiotic stress. In this study, we investigated the interactive effects between salinity (80 and 160 mM NaCl) and exogenous melatonin (100 μM) on the growth, metabolism, and antioxidant capacity of mustard (*Brassica juncea*) sprouts. The results revealed a synergistic interaction in which melatonin effectively mitigated the inhibitory effects of salinity and optimized the balance between growth and defense metabolism. Under moderate salinity, the combined treatment (MN1) significantly enhanced biomass accumulation, soluble sugars, proteins, and glucosinolate retention, while markedly increasing ascorbic acid, total phenolics, and antioxidant capacity. Principal component and membership function analyses confirmed that the melatonin × salinity interaction improved overall physiological performance more effectively than either factor alone. These results demonstrate that melatonin effectively enhances stress resilience and nutritional quality in mustard sprouts, providing a promising strategy for improving the functional value of sprouting vegetables under salinity conditions.

## 1. Introduction

Sprouts are young edible seedlings produced during seed germination and are increasingly valued for their high nutritional content, rapid production cycle, and low cultivation cost [1]. Among them, *Brassica* sprouts are rich in vitamins, phenolics, flavonoids, and glucosinolates, compounds associated with strong antioxidant and health-promoting effects [2,3,4]. Mustard (*Brassica juncea*), a representative *Brassica* species widely cultivated in southwestern China, is inexpensive, locally available, and highly adaptable [5], making it a suitable material for sprout production and functional food development.

Salt stress is traditionally regarded as an abiotic stress factor that disrupts osmotic balance and induces oxidative damage in plants [6]. However, increasing evidence indicates that low concentrations of salt can stimulate growth and enhance the biosynthesis of phytochemicals [7]. For example, mild salinity promotes the synthesis of endogenous growth hormones and improves photosynthesis in broccoli sprouts [8]. Salt elicitation has been successfully used to enhance phytochemical accumulation during germination, such as in durum wheat seedlings [9] and mung bean sprouts [10], where moderate NaCl increased phenolic compounds and antioxidant activity. In rapeseed, salinity stress imposed on parental plants resulted in transgenerational enhancement of phenolic content and antioxidant activity in the offspring sprouts [11]. Furthermore, combinations of salt with other elicitors have shown synergistic effects—for instance, salt solution combined with slightly acidic electrolyzed water promoted millet sprout growth and optimized protein and amino acid profiles [12]. Moderate salt stress also enhanced the enrichment of bioactive compounds in maize sprouts when combined with endogenous selenium [13], and the co-application of methyl jasmonate and salinity increased anthocyanin accumulation in radish sprouts [14]. These findings suggest that moderate salinity, alone or in combination with other treatments, can act as a positive elicitor for improving the nutritional and functional quality of sprouts.

Melatonin (MT), a newly recognized plant hormone, has been widely applied to alleviate abiotic stress-induced damage and also plays important roles in regulating plant growth, development, and quality formation [15]. It not only enhances stress tolerance but also improves morphological traits such as hypocotyl length, cotyledon width, and overall biomass, thereby promoting sprout vigor and development [16,17]. Exogenous melatonin has been reported to increase the accumulation of soluble sugars, polyphenols, and flavonoids, serving as an effective strategy to enhance both growth and the functional food value of quinoa sprouts [18]. Combined treatments, such as slightly acidic conditions with melatonin, have been shown to promote flavonoid biosynthesis in black soybean sprouts [19]. In *Brassica* sprouts, environmental and biochemical elicitors have also been shown to regulate metabolite accumulation and improve nutritional quality. For instance, light quality and temperature were found to jointly modulate the synthesis of key secondary metabolites in broccoli sprouts, where blue or combined red-blue illumination enhanced the accumulation of phenolics, flavonoids, and glucosinolates in cotyledons compared with monochromatic red light [20]. In addition, melatonin elicitation has been reported to enhance antioxidant enzyme activities and delay senescence in horticultural species, such as carnations exposed to simulated transport stress [21]. These studies collectively confirm that both physical and biochemical elicitors can substantially affect plant metabolism and antioxidant defense.

However, the interactive effects of melatonin and moderate salinity on the physiological performance and metabolic regulation of *Brassica* sprouts have not been systematically investigated. Therefore, this study aimed to investigate the combined effects of NaCl and melatonin on the growth, primary metabolism, antioxidant capacity, and glucosinolate accumulation in mustard sprouts. The findings will provide new insights into the physiological and metabolic regulation of sprout quality and offer a potential strategy for improving the nutritional value of *Brassica* sprouts through optimized elicitation.

## 2. Results

### 2.1. Appearance and Growth Performance

**Figure 1:**
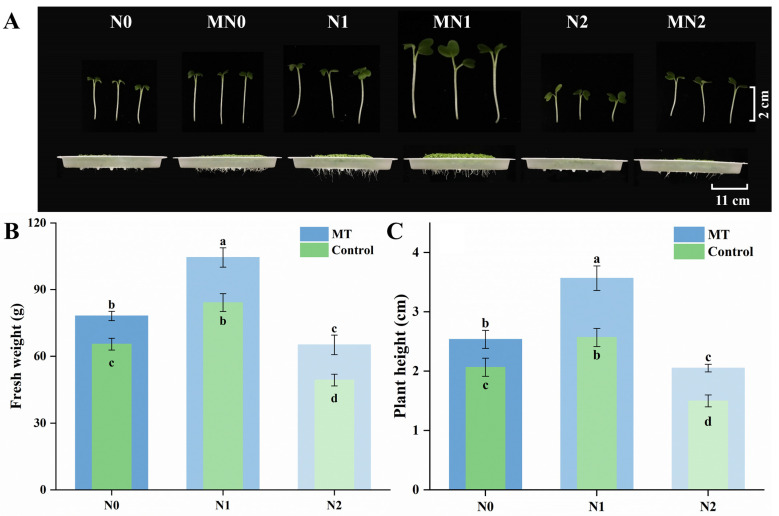
Effects of NaCl and melatonin treatments on the growth performance of mustard sprouts. (**A**) Representative appearance of mustard sprouts under different treatments; (**B**) plant height; (**C**) fresh weight. Different letters in the figure indicate statistically significant differences among treatments according to the least significant difference (LSD) test at *p* < 0.05.

The appearance and growth of mustard sprouts were notably affected by NaCl stress and melatonin treatment (Figure 1A–C). Moderate salinity (N1, 80 mM NaCl) significantly promoted sprout growth, whereas high salinity (N2, 160 mM NaCl) markedly inhibited it. Melatonin application enhanced growth under all conditions (N0, N1, and N2), resulting in more vigorous sprouts (Figure 1A).

Both plant height and fresh weight exhibited similar trends. Compared with the control (N0), plant height increased by 24.2% under N1 treatment but declined by 27.4% under N2 treatment (Figure 1B). When combined with melatonin, the corresponding increases (MN0, MN1, MN2) were 1.23-, 1.39-, and 1.37-fold higher than their respective non-melatonin controls. Fresh weight followed the same pattern: it rose by 28.6% under N1 compared with N0, but decreased by 24.5% under N2 (Figure 1C). With melatonin supplementation, MN0, MN1, and MN2 exhibited 1.19-, 1.24-, and 1.32-fold increases relative to N0, N1, and N2, respectively.

### 2.2. Soluble Sugar and Protein Content

**Figure 2:**
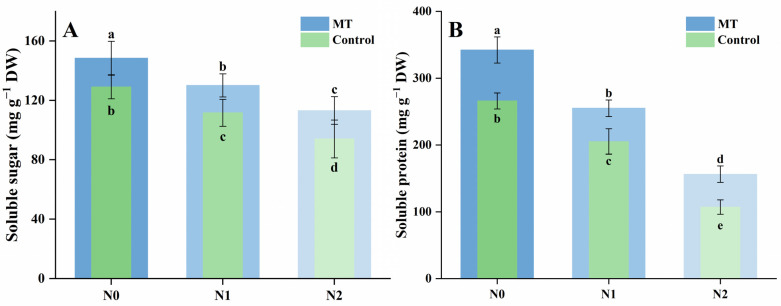
Effects of NaCl and melatonin treatments on soluble sugar and soluble protein content in mustard sprouts. (**A**) Soluble sugar content; (**B**) soluble protein content. Different letters in the figure indicate statistically significant differences among treatments according to the least significant difference (LSD) test at *p* < 0.05.

Soluble sugar and protein levels in mustard sprouts were markedly influenced by NaCl and melatonin treatments (Figure 2A,B). Compared with the control (N0), soluble sugar content decreased by 13.5% under N1 and by 27.1% under N2, indicating that salt stress suppressed carbohydrate accumulation. Melatonin supplementation increased soluble sugar levels under all conditions, with MN0, MN1, and MN2 showing 1.15-, 1.17-, and 1.20-fold higher values than their corresponding controls, respectively.

A similar pattern was observed for soluble protein content. Relative to N0, soluble protein levels declined by 22.8% and 59.8% under N1 and N2, respectively. The addition of melatonin significantly alleviated this decline, with MN0, MN1, and MN2 exhibiting 1.29-, 1.24-, and 1.46-fold increases compared with N0, N1, and N2, respectively.

### 2.3. Ascorbic Acid and Total Phenolic Content

**Figure 3:**
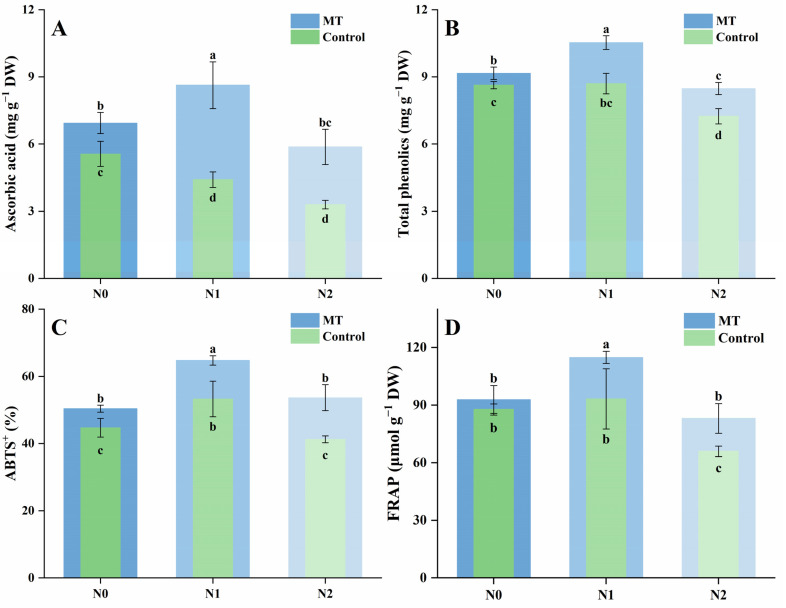
Effects of NaCl and melatonin treatments on antioxidant compounds and antioxidant capacity in mustard sprouts. (**A**) Ascorbic acid content; (**B**) total phenolics content; (**C**) ABTS; (**D**) FRAP. Different letters in the figure indicate statistically significant differences among treatments according to the least significant difference (LSD) test at *p* < 0.05.

Ascorbic acid and total phenolic content of mustard sprouts were significantly affected by NaCl stress and melatonin application (Figure 3A,B). Increasing salinity led to a gradual decline in ascorbic acid content, with reductions of 20.7% and 40.8% under N1 and N2 compared with the control (N0). Melatonin supplementation increased ascorbic acid levels across all conditions, being 1.25-, 1.96-, and 1.78-fold higher in MN0, MN1, and MN2 than in their corresponding N0, N1, and N2 treatments. Among all treatments, MN1 showed the highest ascorbic acid content.

For total phenolics content, N1 caused little change, while N2 reduced the level by 16.1% relative to N0. Melatonin addition enhanced phenolic accumulation under salt stress, with MN1 and MN2 being 1.21- and 1.17-fold higher than N1 and N2, respectively. The highest total phenolic content was also observed in MN1.

### 2.4. Antioxidant Capacity

The antioxidant capacity of mustard sprouts, assessed by ABTS and FRAP, was significantly affected by NaCl and melatonin treatments (Figure 3C,D). For ABTS activity, N1 increased antioxidant capacity by 19.1% compared with N0, while N2 showed no significant difference from N0. Melatonin supplementation enhanced antioxidant activity across all conditions, with MN0, MN1, and MN2 showing 1.13-, 1.22-, and 1.30-fold higher values than their respective non-melatonin treatments. Among all treatments, MN1 exhibited the highest ABTS activity.

For FRAP reducing power, N1 did not differ significantly from N0, whereas high salinity (N2) caused a 24.9% reduction. Melatonin application increased FRAP values under salt stress, with MN1 and MN2 being 1.23- and 1.26-fold higher than N1 and N2, respectively. No significant difference was observed between N0 and MN0. The MN1 treatment showed the highest overall antioxidant capacity, consistent with its elevated levels of ascorbic acid and phenolics. Overall, melatonin notably improved antioxidant capacity under salinity, particularly under moderate salt conditions.

### 2.5. Glucosinolate Composition and Content

**Figure 4:**
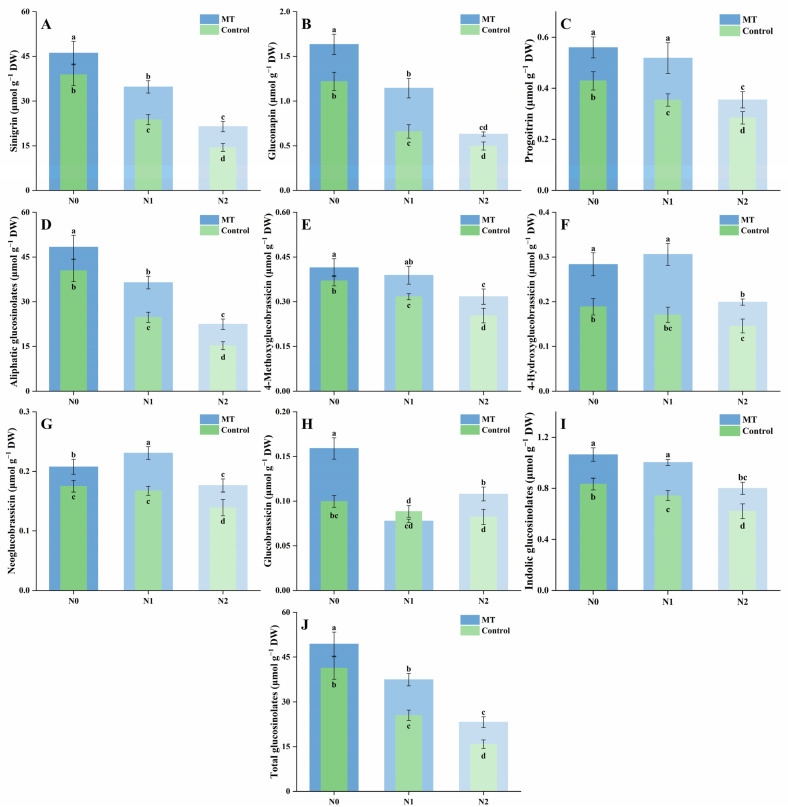
Effects of NaCl and melatonin treatments on glucosinolate profiles and content in mustard sprouts. (**A**) Sinigrin; (**B**) gluconapin; (**C**) progoitrin; (**D**) total aliphatic glucosinolates; (**E**) 4-methoxyglucobrassicin; (**F**) 4-hydroxyglucobrassicin; (**G**) neoglucobrassicin; (**H**) glucobrassicin; (**I**) total indolic glucosinolates; (**J**) total glucosinolates. Different letters in the figure indicate statistically significant differences among treatments according to the least significant difference (LSD) test at *p* < 0.05.

Three aliphatic glucosinolates—sinigrin, gluconapin, and progoitrin—were detected in mustard sprouts (Figure 4A–C). For sinigrin, increasing NaCl concentration significantly reduced its content, with decreases of 38.8% and 62.8% under N1 and N2 treatments compared with N0. Melatonin application elevated sinigrin levels across all conditions, with MN0, MN1, and MN2 being 1.19-, 1.46-, and 1.48-fold higher than their respective controls. For gluconapin, salt stress led to a similar downward trend, with content decreasing by 45.9% and 59.2% under N1 and N2 relative to N0. Melatonin supplementation increased gluconapin accumulation, but N2 and MN2 showed no significant difference. For progoitrin, moderate and high salinity caused declines of 17.5% and 33.7%, respectively, compared with N0. Melatonin treatment increased progoitrin levels, and MN0 and MN1 showed no significant difference, while both were higher than their corresponding salt-only treatments. The total aliphatic glucosinolate content followed the same trend as the individual components (Figure 4D). Compared with N0, total glucosinolates decreased by 38.8% and 62.4% under N1 and N2 treatments, while melatonin application increased the corresponding levels by 1.19-, 1.47-, and 1.47-fold in MN0, MN1, and MN2 relative to N0, N1, and N2, respectively.

Four indolic glucosinolates—4-methoxyglucobrassicin, 4-hydroxyglucobrassicin, neoglucobrassicin, and glucobrassicin—were identified in mustard sprouts, and their total content was calculated (Figure 4E–H). For 4-methoxyglucobrassicin, NaCl stress led to a gradual reduction, with content decreasing by 14.5% and 31.6% under N1 and N2 compared with N0. Melatonin supplementation increased its level in all treatments, with MN0, MN1, and MN2 being 1.12-, 1.23-, and 1.25-fold higher than their corresponding controls. For 4-hydroxyglucobrassicin, the difference between N0 and N1 was not significant, while N2 showed a clear decline of 22.7% relative to N0. Melatonin treatment increased this compound across all salinity levels; MN0 and MN1 were markedly higher than their non-melatonin counterparts, whereas MN2 remained lower but still exceeded N2. For neoglucobrassicin, N0 and N1 showed no significant difference, but N2 decreased by 20.6% compared with N0. Melatonin elevated its accumulation, with MN0, MN1, and MN2 being 1.19-, 1.38-, and 1.27-fold higher than the respective controls. The increase was most pronounced in MN1, while N1 and MN1 were not significantly different. For glucobrassicin, NaCl stress caused a progressive reduction, although N0 and N1 did not differ significantly. The content under N2 declined by 17.5% compared with N0. Melatonin supplementation enhanced glucobrassicin accumulation under all conditions, while N1 and MN1 showed no significant difference. The total indolic glucosinolate content decreased with increasing salinity, by 10.9% and 25.6% under N1 and N2 relative to N0 (Figure 4I). Melatonin application increased total indolic glucosinolates at all salinity levels, with MN0, MN1, and MN2 being 1.28-, 1.35-, and 1.29-fold higher than their respective controls. However, MN0 and MN1 did not differ significantly.

The total glucosinolate content of mustard sprouts showed a clear downward trend with increasing NaCl concentration (Figure 4J). Compared with the control (N0), total glucosinolates decreased by 38.2% and 61.6% under N1 and N2 treatments, respectively. The addition of melatonin significantly increased the total glucosinolate content under all conditions, with MN0, MN1, and MN2 being 1.20-, 1.47-, and 1.47-fold higher than their corresponding N0, N1, and N2 treatments. Among all treatments, MN0 and MN1 maintained relatively high glucosinolate levels, whereas MN2 partially alleviated the reduction caused by high salinity.

### 2.6. Principal Component Analysis

**Figure 5:**
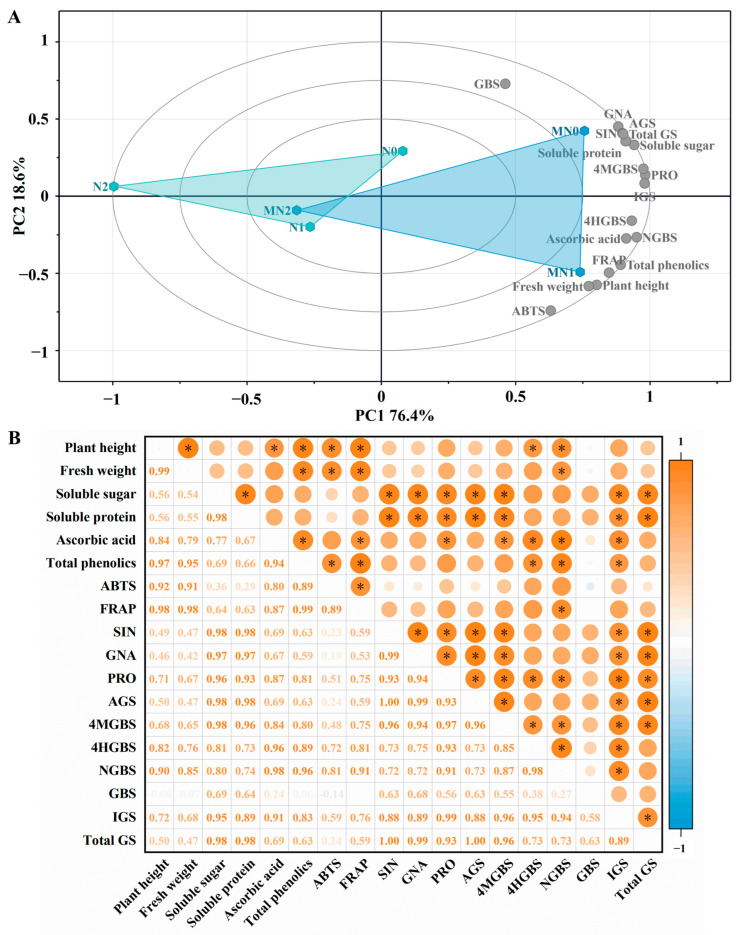
Principal component (**A**) and correlation analysis (**B**) of physiological, antioxidant, and glucosinolate parameters in mustard sprouts under NaCl and melatonin treatments. SIN, Sinigrin; GNA, gluconapin; PRO, progoitrin; AGS, total aliphatic glucosinolates; 4MGBS, 4-methoxyglucobrassicin; 4HGBS, 4-hydroxyglucobrassicin; NGBS, neoglucobrassicin; GBS, glucobrassicin; IGS, total indolic glucosinolates; Total GS, total glucosinolates. * *p* ≤ 0.05. green triangle: control; blue triangle: melatonin treatment.

Principal component analysis was conducted to evaluate the overall effects of NaCl and melatonin treatments on the physiological and biochemical traits of mustard sprouts (Figure 5A). The first two principal components (PC1 and PC2) explained 76.4% and 18.6% of the total variance, respectively, accounting for 95.0% of the cumulative variation. According to the loading scores, PC1 was mainly defined by individual glucosinolates and soluble sugar, while PC2 was primarily associated with ABTS, plant height, fresh weight, and glucobrassicin.

Samples were clearly separated according to treatment type. N0 and MN0 were located in the first quadrant, whereas NaCl-treated groups (N1, MN1, N2, MN2) were distributed across the other three quadrants with greater distances, indicating pronounced effects of salt stress. N0 was closely associated with soluble sugar, soluble protein, and aliphatic glucosinolates, while N1 correlated more strongly with plant height, fresh weight, total phenolics and so on. In contrast, N2 was positioned opposite most quality-related indicators, reflecting its inhibitory effects under high salinity.

Melatonin-treated samples were clearly separated from the corresponding controls, with Control (green triangles) and Melatonin (blue triangles) forming distinct clusters. Among them, MN0 was positioned near soluble sugar, soluble protein, and glucosinolate indicators, whereas MN1 was closely related to growth- and antioxidant-associated parameters. These results indicate that melatonin broadly improved growth and nutritional performance under both normal and salt-stress conditions.

### 2.7. Correlation Analysis

Correlation analysis was performed to examine the relationships among growth parameters, antioxidant properties, and glucosinolate accumulation in mustard sprouts (Figure 5B). Plant height and fresh weight showed significant positive correlations with total phenolic content, antioxidant capacity (ABTS and FRAP), and neoglucobrassicin, indicating that these parameters were closely associated with growth performance. Soluble sugar and soluble protein were positively correlated with most glucosinolates, particularly the aliphatic types.

FRAP exhibited strong positive correlations with ascorbic acid and total phenolics, confirming the close linkage between antioxidant compounds and reducing power. The aliphatic glucosinolates (sinigrin, gluconapin, and progoitrin) were positively correlated with each other, while the indolic glucosinolates displayed more variable relationships. Among them, glucobrassicin showed weak correlations with other indicators. In contrast, total glucosinolate content was positively correlated with soluble sugar, soluble protein, and total aliphatic glucosinolates, reflecting a coordinated variation between primary metabolism and glucosinolate biosynthesis.

### 2.8. Membership Function Analysis and Comprehensive Score

Membership function analysis was used to comprehensively evaluate the effects of NaCl and melatonin treatments on the overall growth and quality of mustard sprouts (Table 1). The comprehensive score for each treatment was calculated based on normalized physiological and biochemical indicators.

Among all treatments, MN0 (0.82) and MN1 (0.80) achieved the highest scores, indicating that melatonin markedly improved the comprehensive performance of mustard sprouts under both normal and moderate salt conditions. N0 (0.50) ranked third, followed by N1 (0.34) and MN2 (0.32), while the lowest score was observed in N2 (0.00), reflecting the strongest inhibitory effect of high salinity. These results suggest that melatonin effectively alleviates salt-induced stress and enhances the overall growth and quality of mustard sprouts.

## 3. Discussion

Salt stress has contrasting effects on plant metabolism depending on its severity, whereas melatonin alleviates salt-induced damage and improves physiological performance [8,15,22]. Here, we investigated their combined influence on the growth and quality of mustard sprouts.

In this study, moderate salinity (80 mM NaCl) significantly promoted the growth of mustard sprouts, whereas high salinity (160 mM NaCl) strongly inhibited elongation and biomass accumulation. Although salt stress is generally considered detrimental to plant growth, similar dual effects have been reported in several sprouting vegetables. For instance, increasing NaCl concentration progressively inhibited sprout length and biomass in pea [23] and peanut sprouts [24], as well as in *Salvia hispanica* sprouts [25], where germination parameters decreased linearly with salinity. However, moderate salt levels (40–80 mM NaCl) significantly stimulated sprout growth in broccoli, while higher concentrations (≥120 mM) suppressed it [8]. Likewise, low concentrations of organic calcium salts enhanced stem elongation and biomass accumulation in common vetch (*Vicia sativa* L.) sprouts [26]. Such promotion under mild salinity may be attributed to the stimulation of osmotic adjustment, photosynthetic efficiency, and hormone signaling balance, as previously observed in broccoli sprouts [8]. Melatonin treatment enhanced sprout growth under both control and salt-stress conditions. Consistent with previous findings, melatonin positively influences germination rate, root elongation, shoot growth, and total biomass in various plant species [16,17]. Under stress conditions, it improves seed vigor, alleviates oxidative damage, and counteracts the inhibition of photosynthesis and metabolism [15,27]. Exogenous melatonin application has been shown to mitigate NaCl-induced growth inhibition in bean [28] and soybean [29] sprouts by increasing shoot length, surface area, volume, and diameter, thereby restoring normal development. In the present study, melatonin effectively alleviated salt-induced suppression and further enhanced growth even under non-stress conditions, confirming its broad regulatory role in maintaining sprout vigor and growth potential.

Soluble sugars and proteins serve as vital energy reserves and osmoprotective compounds in plants [30]. Carbohydrate metabolism plays a crucial role in plant adaptation to abiotic stresses, providing substrates for energy production and maintaining osmotic balance [31,32]. In this study, both moderate and high NaCl concentrations significantly reduced the content of soluble sugar and protein in mustard sprouts. The decline may be attributed to aggravated cellular injury under salinity, including membrane disruption and oxidative imbalance caused by excessive reactive oxygen species (ROS) accumulation [31]. These conditions likely interfere with macromolecular metabolism, leading to the destabilization of carbohydrate biosynthesis and related pathways. In contrast, moderate salt levels have been reported to increase carbohydrate accumulation in *Lepidium sativum* sprouts [33], suggesting that the response to salinity varies among species. Melatonin treatment markedly enhanced soluble sugar and protein accumulation under all salinity conditions, which can be attributed not only to its role as an antioxidant but also to its signaling function in activating ROS-scavenging and osmotic adjustment pathways. Previous studies have shown that melatonin upregulates antioxidant enzyme activities, thereby reducing oxidative stress and stabilizing membrane integrity [34,35]. In addition, melatonin regulates genes involved in osmolyte biosynthesis, including those related to soluble sugar, proline, and protein metabolism, maintaining intracellular osmotic homeostasis [36]. In *Chenopodium quinoa* sprouts [18], melatonin increased total soluble sugar accumulation, while in cotton leaves, it elevated sugar content and enhanced salt resistance [35]. Similarly, melatonin alleviated osmotic stress in *Brassica napus* seedlings by promoting soluble protein synthesis and maintaining osmotic balance [36]. The increase in soluble sugar and protein in melatonin-treated mustard sprouts observed in this study further supports its role as a ROS scavenger and metabolic regulator, helping to preserve membrane integrity, stabilize cellular metabolism, and sustain growth under salt conditions.

Ascorbic acid and total phenolic content did not increase under moderate salinity but significantly declined under high salinity, suggesting that oxidative damage occurred in mustard sprouts at elevated NaCl concentrations. Salt stress has been identified as a potential elicitor that can enhance phytochemical accumulation during germination [9]. For instance, moderate NaCl treatment (<100 mM) improved health-promoting attributes in mung bean sprouts [10], and 150 mM NaCl increased resveratrol content in peanut sprouts [24]. Similarly, salt elicitation promoted phenolics and flavonoid accumulation in *Lepidium sativum* sprouts [33], while DPPH activity was enhanced under 40–80 mM NaCl. However, in the present study, the NaCl concentration applied to mustard sprouts might not have been optimal for elicitation, as excessive ionic stress suppressed the biosynthesis of antioxidant metabolites [37]. Interestingly, ABTS antioxidant capacity increased under moderate salinity, indicating that total antioxidant performance was not solely dependent on ascorbic acid and phenolics. Other antioxidant compounds, such as flavonoids or enzymatic antioxidants, might contribute to this enhancement [38]. In *Brassica* sprouts, salt-induced accumulation of secondary metabolites often reflects a complex balance between oxidative signaling and stress inhibition. The synergistic enhancement observed under melatonin and moderate salinity likely reflects an “elicitor synergy” mechanism, where mild abiotic stress provides a redox signal that activates secondary metabolism, and melatonin amplifies this response by stabilizing ROS homeostasis and promoting antioxidant gene expression. Similar synergistic responses between chemical or physical elicitors under moderate stress have been reported in *Leguminosae* sprouts, leading to elevated phenolics and antioxidant activity [39,40]. Melatonin treatment increased both ascorbic acid and total phenolic content under all salinity conditions, with the highest levels observed in MN1. This suggests that melatonin and moderate salt stress cooperatively act as dual elicitors, stimulating phenylpropanoid metabolism and enhancing redox capacity. Previous studies have shown that exogenous melatonin enhances sprout growth and antioxidant properties by activating the phenylpropanoid biosynthesis pathway in common bean [39], increasing isoflavone levels in soybean sprouts [40], and inducing phenolic acid accumulation in barley seedlings [41]. These results indicate that melatonin not only counteracts oxidative damage but also acts synergistically with moderate abiotic stress to strengthen elicitor-induced antioxidant responses in mustard sprouts.

In the present study, both moderate and high salinity suppressed glucosinolate accumulation in mustard sprouts. Similar results have been reported in other *Brassica* species and sprouting vegetables. The total glucosinolate content in radish sprouts was significantly reduced under 10 and 50 mM NaCl treatments [42], and salt stress negatively affected glucosinolate accumulation in Ethiopian mustard [43]. In rocket (*Eruca sativa*), glucosinolate levels increased under 65 mM NaCl but declined sharply at 130 mM [44], suggesting that the regulatory effect of salinity depends on both concentration and species. In broccoli sprouts, NaCl preferentially enhanced the formation of anti-cancer isothiocyanates rather than glucosinolates, indicating enhanced degradation or metabolic conversion under salt stress [45]. The overall reduction in glucosinolate content observed in this study might therefore result from decreased soluble sugar availability—limiting precursor supply—and increased degradation rates under oxidative conditions. Melatonin, a newly recognized plant growth regulator, not only mitigates abiotic stress damage but also promotes the biosynthesis of secondary metabolites [46]. In the current experiment, melatonin treatment increased glucosinolate accumulation under all salinity conditions. Similar effects were observed in broccoli, where melatonin upregulated the expression of glucosinolate biosynthetic genes and led to higher total glucosinolate content [47]. This enhancement may be related to improved substrate availability and transcriptional activation of key enzymes in the biosynthetic pathway. Collectively, these findings indicate that melatonin alleviates salt-induced suppression of glucosinolate metabolism and facilitates the coordinated regulation of stress defense and secondary metabolite production in mustard sprouts.

The combination of principal component and membership function analyses provided an integrated perspective on the overall physiological performance of mustard sprouts under different NaCl and melatonin treatments. The principal component analysis (PCA) clearly distinguished the treatments, revealing that salinity and melatonin induced distinct metabolic adjustments. Variables related to growth and antioxidant activity were mainly associated with moderate salinity, whereas those linked to carbohydrate and glucosinolate metabolism were clustered with the melatonin treatments, indicating that melatonin rebalanced primary and secondary metabolism under stress. The comprehensive evaluation further confirmed that melatonin significantly improved the overall quality of mustard sprouts, regardless of salinity. Among all treatments, the melatonin-supplemented groups exhibited consistently higher integrated scores, reflecting simultaneous promotion of growth, nutrient accumulation, and antioxidant capacity. Notably, melatonin combined with moderate salinity produced a complementary effect, enhancing physiological activity without compromising metabolite accumulation. This coordinated improvement implies that melatonin not only mitigates salt-induced inhibition but also modulates carbon allocation and secondary metabolism to optimize sprout development.

From a practical perspective, melatonin application shows strong potential for integration into commercial sprout production systems. Seed priming or co-treatment with melatonin represents a simple and low-cost approach that can be readily implemented in hydroponic or vertical-farming units without major technological modification. Melatonin is a naturally occurring and non-toxic compound that has been recognized as safe for human consumption, and its regulatory acceptance in food and agricultural applications continues to expand. These attributes make it suitable for large-scale use to improve the nutritional and functional quality of sprouts under variable water or salinity conditions. The present findings also suggest that the melatonin-mediated enhancement of primary and secondary metabolism could be transferable to other *Brassica* and microgreen species with comparable biosynthetic pathways, such as *Brassica oleracea* and *Eruca sativa*. Given their shared glucosinolate–phenylpropanoid networks, the combined elicitation strategy of melatonin and moderate abiotic stress may provide a general framework for improving the health-promoting potential of diverse sprouting vegetables.

## 4. Materials and Methods

### 4.1. Plant Materials and Treatments

Seeds of the mustard (*Brassica juncea*) cultivar ‘Chuanbao 11’ were used in this study. Approximately 12 g of seeds were immersed in 40 mL of either distilled water or 100 μM melatonin for 24 h at room temperature. The pretreated seeds were then evenly distributed in plastic seedling trays (33 cm × 26 cm × 4.5 cm) containing 500 mL of distilled water for hydroponic cultivation. Each treatment consisted of three independent trays, which served as biological replicates (experimental units). Germination was initiated in darkness for 2 days, followed by exposure to light under controlled environmental conditions (light intensity 80 μmol m^−2^ s^−1^, temperature 23 °C, photoperiod 16 h light/8 h dark, and relative humidity 70%).

**Figure 6:**
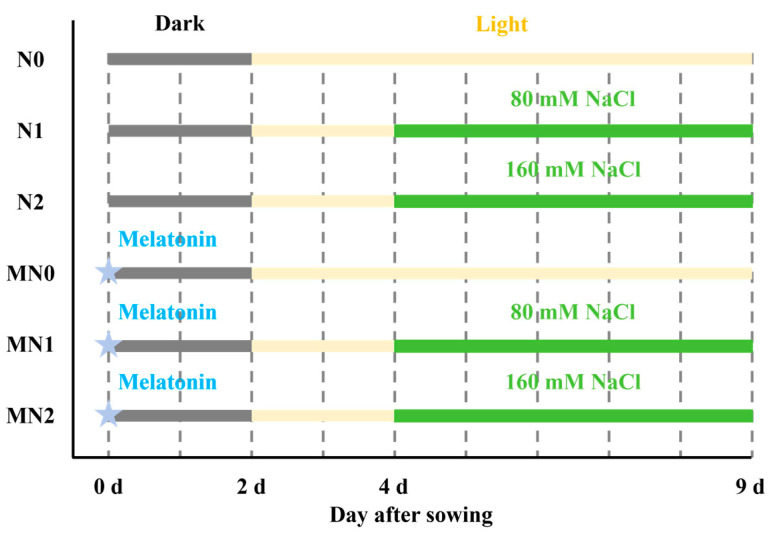
Schematic illustration of the experimental timeline and treatment design.

On the fourth day, sprouts were treated with NaCl solutions at concentrations of 80 mM or 160 mM, while control groups received distilled water. The nutrient solutions were renewed every other day. Six treatment groups were established: distilled water (N0), melatonin alone (MN0), 80 mM NaCl (N1), 160 mM NaCl (N2), melatonin + 80 mM NaCl (MN1), and melatonin + 160 mM NaCl (MN2) (Figure 6).

After nine days of cultivation (corresponding to the sprouting stage with fully expanded cotyledons and no visible true leaves), sprouts were harvested by excising them at the root base. Samples designated for transcriptomic analysis were immediately frozen in liquid nitrogen and stored at −80 °C. The remaining samples were freeze-dried (EYELA FDU-2110, Tokyo, Japan), ground into fine powder, and stored at −20 °C for subsequent physiological and biochemical assays.

### 4.2. Growth Parameters

Thirty mustard sprouts were randomly selected for each treatment, with ten plants per replicate. Each treatment included three biological replicates corresponding to three independent plastic seedling trays (experimental units). Plant height was measured from ten randomly chosen sprouts per tray using a ruler, while fresh weight was determined based on the total aboveground biomass harvested from each tray after removing roots, using an electronic balance [16].

### 4.3. Soluble Sugar Content

Dried sample powder (20 mg) was placed in a 10 mL centrifuge tube, extracted with 8 mL distilled water at 90 °C for 20 min, and centrifuged at 4000× *g* for 5 min. Then, 1 mL of supernatant was mixed with 0.5 mL anthrone–ethyl acetate reagent and 5 mL concentrated sulfuric acid, incubated at 90 °C for 5 min, and the absorbance was read at 630 nm within 5 min [48].

### 4.4. Soluble Protein Content

Twenty milligrams of dried powder were extracted with 8 mL of distilled water, vortexed for 30 s, allowed to stand for 40 min, and centrifuged at 4000× *g* for 5 min. One milliliter of supernatant was mixed with 5 mL Coomassie brilliant blue G-250 solution, and the absorbance was determined at 595 nm within 20 min [48].

### 4.5. Ascorbic Acid Content

Ascorbic acid was extracted from 50 mg of sample powder using 5 mL of 1.0% (*w*/*v*) oxalic acid, followed by centrifugation (4000× *g*, 5 min) and filtration through a 0.45 μm membrane. The filtrate (20 μL) was analyzed on a Waters Spherisorb C18 column (250 × 4.6 mm, 5 μm; Waters Corporation, Milford, MA, USA) with 0.1% oxalic acid as the mobile phase at 1.0 mL min^−1^. Detection was performed at 243 nm, and quantification was based on an ascorbic acid standard curve [48].

### 4.6. Total Phenolics Content

Powdered samples (40 mg) were extracted with 50% ethanol and centrifuged at 4000× *g* for 5 min. The supernatant (300 μL) was mixed with 1.5 mL of 0.2 mol L^−1^ Folin–Ciocalteu reagent and incubated for 3 min in the dark, followed by the addition of 1.2 mL saturated sodium carbonate. After 20 min at room temperature, absorbance was measured at 760 nm. Gallic acid was used as the calibration standard [48].

### 4.7. 2,2-Azinobis (3-Ethyl-benzothiazoline-6-sulfonic Acid) (ABTS^+^) Assay

ABTS^+^ radicals were produced by reacting 7 mM ABTS solution with 2.45 mM ammonium persulfate and allowing it to stand in darkness for 16 h. The mixture was diluted with acetate buffer (pH 4.5) to an absorbance of 0.700 ± 0.020 at 734 nm. Then, 300 μL of each extract was added to 3 mL of the ABTS^+^ solution. After 2 h of reaction, absorbance was read at 734 nm, and radical scavenging activity was calculated based on inhibition percentage [48].

### 4.8. Ferric Reducing Antioxidant Power (FRAP)

The FRAP reagent consisted of 300 mM acetate buffer (pH 3.6), 20 mM ferric chloride, and 10 mM 2,4,6-tripyridyl-s-triazine in 40 mM HCl mixed at a ratio of 10:1:1 (*v*/*v*/*v*). Extracted samples (300 μL) were combined with 2.7 mL of FRAP reagent, incubated at 37 °C for 10 min, and measured at 593 nm. Results were expressed as μmol g^−1^ dry weight, using FeSO_4_·7H_2_O as a standard [48].

### 4.9. Glucosinolate Content

Dry samples (100 mg) were extracted twice with 5 mL boiling distilled water for 10 min and centrifuged at 4000× *g* for 5 min. The combined supernatants (1 mL) were loaded onto DEAE-Sephadex A-25 columns (Sigma Chemical Co., St. Louis, MI, USA), desulfated overnight with 100 μL of 0.1% aryl sulfatase, and eluted with 1 mL water. Desulfo-glucosinolates were analyzed by HPLC (Agilent 1260, VWD detector; Agilent Technologies, Inc., Palo Alto, CA, USA) on a Waters Spherisorb C18 column (250 × 4.6 mm, 5 μm; Waters Corporation, Milford, MA, USA) at 30 °C, using acetonitrile and water as mobile phases (1.0 mL min^−1^). Detection was performed at 226 nm, and quantification was based on ortho-nitrophenyl β-D-galactopyranoside as internal standard with respective response factors [48].

### 4.10. Statistical Analysis

All data were expressed as mean ± standard deviation (SD). Prior to analysis of variance (ANOVA), data were tested for normality using the Shapiro–Wilk test and for homogeneity of variance using Levene’s test. Statistical significance among treatments was determined by two-way ANOVA, and mean comparisons were performed using the least significant difference (LSD) test at *p* < 0.05. All statistical analyses were conducted with DPS 9.01. Figures were prepared using OriginPro 2024 (OriginLab, Northampton, MA, USA), and PCA was carried out with SIMCA 14.1 (Umetrics, Malmö, Sweden).

A comprehensive evaluation of growth and quality indices was conducted using the membership function approach in fuzzy mathematics, as described by Yan et al. [49]. Positively correlated indicators were normalized based on their maximum and minimum values, while negatively correlated indicators were inversely transformed to ensure consistent scoring across all parameters.

## 5. Conclusions

Moderate salinity exerted a mild elicitation effect that stimulated metabolic activity and antioxidant potential in mustard sprouts, whereas high salinity caused pronounced inhibition. Melatonin further enhanced these beneficial effects and alleviated salt-induced damage by maintaining redox balance and coordinating primary and secondary metabolism. From an application perspective, melatonin seed priming or co-treatment offers a simple, safe, and low-cost strategy that can be readily applied in hydroponic or vertical sprout production systems. This combined elicitation approach may also be transferable to other Brassica and microgreen species, providing a sustainable means to enhance the functional and nutritional value of sprouting vegetables under moderate environmental stress.

## Figures and Tables

**Table 1:** A comprehensive evaluation of the effects of combined treatments of NaCl and melatonin on the growth and quality indicators of mustard sprouts.

Treatments	Plant Height	Fresh Weight	Soluble Sugar	Soluble Protein	Ascorbic Acid	Total Phenolics
N0	0.27	0.29	0.64	0.68	0.43	0.42
N1	0.52	0.63	0.32	0.42	0.21	0.44
N2	0.00	0.00	0.00	0.00	0.00	0.00
MN0	0.50	0.52	1.00	1.00	0.68	0.58
MN1	1.00	1.00	0.66	0.63	1.00	1.00
MN2	0.27	0.29	0.35	0.21	0.48	0.38
**Treatments**	**ABTS**	**FRAP**	**AGS**	**IGS**	**Comprehensive** **evaluation value**	**Rank**
N0	0.15	0.45	0.76	0.48	0.50	3
N1	0.51	0.56	0.29	0.28	0.34	4
N2	0.00	0.00	0.00	0.00	0.00	6
MN0	0.39	0.55	1.00	1.00	0.82	1
MN1	1.00	1.00	0.64	0.86	0.80	2
MN2	0.53	0.35	0.22	0.41	0.32	5

## Data Availability

The original contributions presented in this study are included in the article material. Further inquiries can be directed to the corresponding authors.
